# Decline in cognitively complex everyday activities accelerates along the Alzheimer’s disease continuum

**DOI:** 10.1186/s13195-020-00706-2

**Published:** 2020-10-29

**Authors:** Mark A. Dubbelman, Roos J. Jutten, Sarah E. Tomaszewski Farias, Rebecca E. Amariglio, Rachel F. Buckley, Pieter Jelle Visser, Dorene M. Rentz, Keith A. Johnson, Michael J. Properzi, Aaron Schultz, Nancy Donovan, Jennifer R. Gatchell, Charlotte E. Teunissen, Bart N. M. Van Berckel, Wiesje M. Van der Flier, Reisa A. Sperling, Kathryn V. Papp, Philip Scheltens, Gad A. Marshall, Sietske A. M. Sikkes, for the Alzheimer Disease Neuroimaging Initiative, National Alzheimer’s Coordinating Center, the Harvard Aging Brain Study, the Alzheimer Dementia Cohort

**Affiliations:** 1 Department of Neurology, Amsterdam Neuroscience, Alzheimer Center Amsterdam, Vrije Universiteit Amsterdam, Amsterdam UMC, location VUmc, P.O. Box 7057, 1007 MB Amsterdam, The Netherlands; 2 Department of Neurology, University of California, Davis, Davis, CA USA; 3 Department of Neurology, Massachusetts General Hospital, Harvard Medical School, Boston, MA USA; 4 Department of Neurology, Brigham and Women’s Hospital, Harvard Medical School, Boston, MA USA; 5 Melbourne School of Psychological Sciences, University of Melbourne, Melbourne, Victoria Australia; 6 Department of Psychiatry and Neuropsychology, School for Mental Health and Neuroscience, Maastricht University, Maastricht, The Netherlands; 7 Department of Radiology, Massachusetts General Hospital, Harvard Medical School, Boston, MA USA; 8 Department of Psychiatry, Brigham and Women’s Hospital, Harvard Medical School, Boston, MA USA; 9 Department of Psychiatry, Massachusetts General Hospital, Harvard Medical School, Boston, MA USA; 10 Neurochemistry Laboratory, Department of Clinical Chemistry, Amsterdam UMC, Vrije Universiteit Amsterdam, Amsterdam, The Netherlands; 11 Department of Radiology and Nuclear Medicine, Amsterdam UMC, Vrije Universiteit Amsterdam, Amsterdam, The Netherlands; 12 Department of Clinical, Neuro- & Developmental Psychology, Vrije Universiteit Amsterdam, Amsterdam, The Netherlands

**Keywords:** Alzheimer’s disease, functional impairment, instrumental activities of daily living, clinical stages

## Abstract

**Background:**

Impairment in daily functioning is a clinical hallmark of dementia. Difficulties with “instrumental activities of daily living” (IADL) seem to increase gradually over the course of Alzheimer’s disease (AD), before dementia onset. However, it is currently not well established how difficulties develop along the preclinical and prodromal stages of AD. We aimed to investigate the trajectories of decline in IADL performance, as reported by a study partner, along the early stages of AD.

**Methods:**

In a longitudinal multicenter study, combining data from community-based and memory clinic cohorts, we included 1555 individuals (mean age 72.5 ± 7.8 years; 50% female) based on availability of amyloid biomarkers, longitudinal IADL data, and clinical information at baseline. Median follow-up duration was 2.1 years. All amyloid-positive participants (*n* = 982) were classified into the National Institute on Aging–Alzheimer’s Association (NIA-AA) clinical stages ranging from preclinical AD (1) to overt dementia (4+). Cognitively normal amyloid-negative individuals (*n* = 573) served as a comparison group. The total scores of three study-partner reported IADL questionnaires were standardized.

**Results:**

The rate of decline in cognitively normal (stage 1) individuals with and without abnormal amyloid did not differ (*p* = .453). However, from stage 2 onwards, decline was significantly faster in individuals on the AD continuum (*B [95%CI]* = − 0.32 [− 0.55, − 0.09], *p* = .007). The rate of decline increased with each successive stage: one standard deviation (SD) unit per year in stage 3 (− 1.06 [− 1.27, − 0.85], *p* < .001) and nearly two SD units per year in stage 4+ (1.93 [− 2.19, − 1.67], *p* < .001). Overall, results were similar between community-based and memory clinic study cohorts.

**Conclusions:**

Our results suggest that the rate of functional decline accelerates along the AD continuum, as shown by steeper rates of decline in each successive NIA-AA clinical stage. These results imply that incremental changes in function are a meaningful measure for early disease monitoring. Combined with the low-cost assessment, this advocates the use of these functional questionnaires for capturing the effects of early AD-related cognitive decline on daily life.

## Background

Alzheimer’s disease (AD) pathology, consisting of amyloid-beta plaques, tau neurofibrillary tangles, and neurodegeneration, develops for numerous years before leading to hallmark clinical signs of cognitive and functional impairment [1]. The earliest clinical signs of AD appear to be the subjective experience of memory decline [2, 3], followed by subtle changes in higher-order cognitive functioning. The effects of cognitive decline in daily life can be captured in the performance of cognitively complex “instrumental activities of daily living” (IADL), such as cooking, managing personal and financial paperwork, and keeping appointments.

Impairment in daily functioning is traditionally described as occurring relatively late in the AD disease trajectory, i.e., as a core characteristic of dementia. However, increasing evidence demonstrates that everyday functioning declines gradually over the years preceding the clinical diagnosis of dementia. This has been shown in a number of cross-sectional studies [4–12], as well as a few longitudinal studies [13–17]. Measuring IADL functioning is important, as it is a clinically relevant outcome measure [18], affecting not only the patient, but also their support system by increasing financial [19] and caregiver burden [20]. Moreover, IADL measures have strong ecological validity and they are related directly to daily life. As such, they are valuable in both clinical practice and research.

Individuals with abnormal amyloid are in the Alzheimer’s continuum, and they can be classified into six stages based on clinical symptom severity, according to the U.S. National Institute on Aging–Alzheimer’s Association (NIA-AA) research framework [1]. People in stage 1 (the preclinical phase) do not report a decline in cognition and perform normally on cognitive tests. Stages 2 and 3 (the prodromal phase) are characterized by a self-reported decline in cognition but normal performance on cognitive tests, and the emergence of the first objectified cognitive impairments, respectively. Stages 4 through 6 represent overt dementia with increasing severity of both cognitive and functional impairment. The question remains at what point along the disease trajectory changes in IADL functioning actually start to occur, and how this decline in function develops along the AD continuum.

In this study, we focus especially on the earliest stages (1–3), as we hypothesize that a decline in function may already be present here. We aimed to determine how IADL functioning progresses along the AD continuum, as well as to identify the stage in which decline in functioning is accelerated compared to amyloid-negative, cognitively normal controls. Finally, we aimed to investigate specific activities in more detail, to determine whether there were any differences in the advent of problems and rate of decline in relatively easy and relatively complex IADLs.

## Methods

### Study cohorts and selection criteria

We selected subjects from six cohorts: Harvard Aging Brain Study (HABS, *n* = 259), Alzheimer’s Disease Neuroimaging Initiative (ADNI, *n* = 829), National Alzheimer’s Coordinating Center (NACC, *n* = 201), Amsterdam Dementia Cohort (ADC, *n* = 178), European Medical Information Framework (EMIF)-AD PreclinAD Study (*n* = 73), and EMIF-AD 90+ Study (*n* = 15).

Specific procedures have been described for each cohort in detail elsewhere [21–26]. Briefly, HABS is a prospective community-based cohort that consists of individuals aged 65 years and older, who are considered cognitively normal at study inclusion [21]. ADNI is a multicenter longitudinal cohort study. For the present study, we obtained baseline and follow-up data acquired for ADNI-GO and ADNI-2 [22]. The NACC database contains mostly memory-clinic referred subjects with additional community recruitment [23]. The ADC is a memory-clinic cohort comprised of patients of the Alzheimer Center Amsterdam [24]. In the EMIF-AD PreclinAD Study, cognitively normal subjects aged 60 years and older were included [25]. The EMIF-AD 90+ Study focused on people aged 90 years and older who were either cognitively normal or who had some cognitive impairment [26].

For the present study, subjects were selected based on (1) availability of amyloid biomarkers at baseline, (2) sufficient information to determine NIA-AA clinical staging at baseline, and (3) availability of longitudinal IADL data, defined as having at least one follow-up assessment. All data used in this study were collected between June 2002 and July 2019.

All studies were approved by ethical review boards, and all subjects provided written informed consent for the use of their data for research purposes, in accordance with the Declaration of Helsinki.

### Amyloid

Amyloid status was assessed at baseline using either amyloid positron emission tomography (PET) imaging or cerebrospinal fluid (CSF) using local procedures, such as described in more detail elsewhere [25–30]. PET scans, using ^11^C-Pittsburgh compound-B (PiB) in HABS, ^18^F-florbetapir in ADNI, and one of ^11^C-PiB, ^18^F-flutemetamol, ^18^F-florbetapir, or ^18^F-florbetaben in the ADC and EMIF cohorts, were judged either using standard uptake volume ratios (ADNI, NACC), distribution volume ratios (HABS), or visual rating by independent nuclear medicine physicians (ADC, both EMIF studies). For CSF, local cutoffs for amyloid positivity were used (NACC, ADC). Where both PET and CSF were available for the same individual (*n* = 66), PET results were favored. Both amyloid-positive and amyloid-negative individuals were included. Additional details about amyloid assessment can be found in the Supplementary Material.

### Clinical stages

Amyloid-positive individuals were categorized into four clinical stages according to the NIA-AA framework [1], based on baseline measures of subjective cognitive complaints, cognitive performance, and global functional impairment. This procedure and the measures used are described in detail by Jutten et al. [31]. Briefly, we considered a visit to a memory clinic, or a positive response to a subjective cognitive decline questionnaire as an indication of subjective complaints. Cognitive performance was determined using the scores on a general cognitive screener and a story or list learning task. Finally, functional impairment was determined using a global dementia rating scale. The IADL instruments used as outcomes were not used to determine the stages. Baseline stages are defined as follows: (1) no complaints and no cognitive deficits, objectified using standard neuropsychological testing; (2) subjective complaints but no objectified cognitive deficits; (3) mild objectified cognitive deficits; and (4+) clinically manifest dementia. We did not distinguish between the NIA-AA stages 4, 5 and 6, as the focus of the current investigation was on the preclinical [1] and prodromal stages [2, 3].

Cognitively normal amyloid-negative individuals without cognitive complaints or objectified deficits were included as available from the same cohorts, as a comparison group.

### IADL measures

Three study partner-reported IADL instruments were used: the Functional Activities Questionnaire (FAQ), Everyday Cognition (ECog), and Amsterdam IADL Questionnaire (A-IADL-Q).

The FAQ is a 10-item scale [32]. Each item is rated from 0 (no difficulty or independent) to 3 (dependent), as compared to performance 1 month earlier. We summed all items to compute a total score, ranging from 0 to 30, with higher scores indicating more functional dependence. The ECog is a questionnaire comprised of 39 items reflecting cognitively complex everyday activities across 6 subscales, including memory, language, and executive functioning [33, 34]. All items are rated from 1 (no change in function compared to 10 years ago) to 4 (consistently much worse function than 10 years ago). Total scores are a weighted average ranging from 1 to 4, with higher scores indicating more problems in everyday functioning. The A-IADL-Q is aimed at assessing cognitively complex, relevant everyday activities [35]. It has been extensively validated [36–40]. Item scores range from 0 (no difficulty performing the activity) to 4 (unable to perform the activity), comparing current performance to the past. Total scores are calculated using item response theory and have a mean score of 50 with a standard deviation (SD) of 10 in a memory-clinic population. Higher scores indicate better functioning.

#### Harmonizing IADL measurements

FAQ and ECog raw total scores were inverted so that higher scores represent better functioning. Individual instrument total scores were converted to *Z*-scores using the baseline mean and SD of the entire amyloid-negative subsample. Next, a single *Z*-score was created by pooling the individual instrument *Z*-scores into one. In instances where individuals had both a completed FAQ and ECog, we first averaged the *Z*-scores of the FAQ and ECog, before combining them into the final *Z*-score. The final IADL *Z*-score is thus a standardized measure of IADL performance, with higher scores representing better functioning. A one-unit difference in the IADL *Z*-score represents a change of one SD in functioning among cognitively normal, amyloid-negative individuals.

Furthermore, we harmonized items that referenced the same activities and were shared between the instruments. To illustrate how specific IADLs develop over time, we selected two of these activities on opposite ends on the spectrum of IADL complexity: one relatively easy item (“preparing hot beverages”), and one relatively complex item (“managing the paperwork”). The selection was made a priori on the basis of A-IADL-Q item parameters, as presented by Jutten et al. [37]. The easier item may not be impaired until a relatively high level of overall IADL impairment has been reached, whereas the more complex item may already be impaired at a lower overall level of IADL impairment. The harmonized items are shown in Table 1.

**Table 1:** Harmonization of items and response options from the FAQ, ECog, and A-IADL-Q

Harmonization	FAQ	ECog	A-IADL-Q
Cohort(s)	ADNI, NACC	ADNI, HABS	ADC, EMIF pre-AD and 90+
Item content			
Hot beverages	Heating water, making a cup of coffee, turning off the stove	–	Using the coffee maker
Paperwork	Assembling tax records, business affairs, or other papers	Keeping financial records organized	Managing their household paperwork
Response options			
Normal (4)	Normal (0)	Better or no change (1)	No more difficult (0)
Slightly worse (3)	Has difficulty, but does by self (1)	Questionable/occasionally worse (2)	Slightly more difficult (1)
Worse (2)	–	Consistently a little worse (3)	More difficult (2)
Much worse (1)	Requires assistance (2)	–	Much more difficult (3)
Unable (0)	Dependent (3)	Consistently much worse (4)	No longer able to perform this task (4)

*Abbreviations: ADC* Amsterdam Dementia Cohort, *ADNI* Alzheimer’s Disease Neuroimaging Initiative, *A-IADL-Q* Amsterdam Instrumental Activities of Daily Living Questionnaire, *ECog* Everyday Cognition, *EMIF* European Medical Information Framework, *FAQ* Functional Activities Questionnaire, *HABS* Harvard Aging Brain Study, *NACC* National Alzheimer’s Coordinating Center

### Statistical analyses

Linear or logistic regressions were used to investigate baseline group differences between the amyloid-negative group and each of the four NIA-AA stages. Significance was set at *p* < 0.01. To analyze change over time in IADL functioning, linear mixed models (LMMs) with random intercepts and slopes were run using the “lme4” package version 1.1-27 [41] for R. LMMs are a powerful method for analyzing change over time when handling unbalanced data, including inconsistent time intervals between follow-up measurements and missing data [41]. We fitted models in which the IADL *Z*-score was the dependent variable, and time in years was the main independent variable. Interactions between stage and time were included to determine slopes for each stage, treating the amyloid-negative group as “stage 0” for convenience. Adjustments for clustering within study cohorts, as well as for age at baseline, sex, and education, were also included. Unstandardized estimates and 95% confidence intervals are reported for fixed effects. Finally, we ran sensitivity analyses to investigate potential differences between community-based and memory clinic studies, as well as the influence of each cohort. We ran ordinal logistic mixed-effects models on the two activities, similar to the main analyses. All analyses were run in R version 4.0.2 [42] and Stata version 14 [43].

## Results

### Sample characteristics

A total of 1555 individuals were included (age 72.5 ± 7.8 years old; 49.8% female), of whom 982 were amyloid positive. Mean age did not differ between amyloid-positive and amyloid-negative individuals (*p* = .619). Amyloid-positive individuals had received fewer years of education and had lower MMSE scores at baseline (both *p* < .001) than amyloid-negative individuals. Table 2 displays the baseline characteristics of the amyloid-negative and amyloid-positive groups. Characteristics per cohort can be found in the Supplementary Material.

**Table 2:** Baseline demographics

	Total group (***n*** = 1555)	Amyloid negative (***n*** = 573)	Amyloid positive (***n*** = 982)	Stage 1 (***n*** = 120)	Stage 2 (***n*** = 160)	Stage 3 (***n*** = 464)	Stage 4+ (***n*** = 238)	Post hoc group differences
**Years of follow-up, mean (range)**	2.79 (0.25–7.09)	3.31 (0.46–7.07)	2.49 (0.25–7.09)	2.94 (0.56–6.38)	2.96 (0.46–7.06)	2.69 (0.25–7.09)	1.36 (0.26–4.45)	Amyloid negative, stages 1, 2 > 3 > 4
**Age**	72.45 ± 7.8	72.82 ± 7.1	72.24 ± 8.2	74.72 ± 6.5	74.19 ± 7.7	71.79 ± 8.0	70.55 ± 9.1	Stage 1, 2 > amyloid negative > stages 3, 4
**Female, n (%)**	763 (50)	311 (55)	452 (47)	66 (56)	84 (54)	196 (43)	106 (45)	Amyloid negative, stages 4, 2, 1 > 3
**Education years**	15.37 ± 3.3	15.94 ± 3.1	15.05 ± 3.4	15.94 ± 3.2	15.68 ± 3.2	15.13 ± 3.3	14.01 ± 3.7	Amyloid negative, stage 1, 2 > 3 > 4
**MMSE**	27.08 ± 3.8	29.11 ± 1.1	25.62 ± 4.3	29.06 ± 1.0	28.45 ± 1.7	26.43 ± 2.7	20.08 ± 4.2	Amyloid negative, stage 1 > 2 > 3 > 4
**Cohorts,** ***n*** **(%)**								
HABS	259 (17)	194 (34)	65 (6)	38 (31)	27 (17)	–	–	
ADNI	829 (53)	323 (55)	506 (52)	49 (42)	72 (45)	288 (62)	98 (41)	
NACC	201 (13)	–	201 (21)	29 (25)	23 (14)	84 (18)	65 (27)	
ADC	178 (11)	–	178 (18)	–	15 (9)	87 (19)	76 (32)	
EMIF-AD	73 (5)	53 (9)	20 (2)	2 (2)	18 (11)	–	–	
EMIF-90+	15 (1)	3 (1)	12 (1)	2 (2)	5 (3)	5 (1)	–	

All are displayed as mean ± standard deviation, except as stated otherwise. Missing: Gender (*n* = 22), education (*n* = 13), MMSE (*n* = 192), age (*n* = 14). Group differences between amyloid negative and each of the four NIA-AA stages are based on linear (for all but gender) or logistic (for gender) regression

*Abbreviations: ADC* Amsterdam Dementia Cohort, *ADNI* Alzheimer’s Disease Neuroimaging Initiative, *EMIF* European Medical Information Framework, *HABS* Harvard Aging Brain Study, *MMSE* Mini-Mental State Examination, *NACC* National Alzheimer’s Coordinating Center

All amyloid-positive individuals were classified into one of the NIA-AA clinical stages at baseline: 120 individuals (12%) were in stage 1, 160 (16%) in stage 2, 464 (47%) in stage 3, and the remaining 238 (24%) in stage 4+. Individuals in stages 1 and 2 were older than those in stages 3 and 4, had more years of education, and were more likely to be female (Table 2).

### Overall IADL functioning trajectories

At baseline, 1077 participants completed the ECog, 1025 completed the FAQ, and 266 completed the A-IADL-Q. The correlation between the ECog and FAQ was *r* = .83 (95% confidence interval = [.81, .85], *n* = 819).

At baseline, amyloid-negative (mean (M) ± standard deviation (SD) = 0.05 ± 0.9) and amyloid-positive individuals in stage 1 (0.18 ± 0.6) had similar levels of IADL functioning, on average (*p* = .631). Those in stages 2 (− 0.60 ± 1.6), 3 (− 3.76 ± 3.3), and 4+ (− 8.75 ± 4.3) each had lower baseline functioning. IADL functioning remained fairly stable over time in cognitively normal amyloid-negative individuals (*B* = − 0.08, 95%CI = [− 0.28, 0.14], *p* = .453). In contrast, a substantial decline in IADL functioning was found in the amyloid-positive group as a whole (− 0.95, 95%CI = [− 1.20, − 0.69], *p* < .001; see Table 3).

**Table 3:** Linear mixed model results of change over time in IADL functioning at baseline for amyloid negatives and amyloid positives, divided into the NIA-AA stages

Groups	***B***	95%CI	***P*** value
**Intercepts**			
Amyloid negative	− 1.48	[− 3.46, 0.31]	—^a^
Amyloid positive	− 7.31	[−9.88, − 4.74]	< .001^a^
Stage 1	− 0.80	[− 2.64, 1.04]	.631^a^
Stage 2	− 1.65	[− 3.48, 0.17]	.005^a^
Stage 3	− 4.67	[− 6.43, − 2.92]	< .001^a^
Stage 4+	− 9.64	[− 11.41, − 7.88]	< .001^a^
**Slopes**			
Amyloid negative	− 0.08	[− 0.28, 0.14]	.453
Amyloid positive	− 0.94	[− 1.20, − 0.69]	< .001
Stage 1	− 0.12	[− 0.37, 0.13]	.342
Stage 2	− 0.32	[− 0.55, − 0.09]	.007
Stage 3	− 1.06	[− 1.27, − 0.85]	< .001
Stage 4+	− 1.93	[− 2.19, − 1.67]	< .001

Shown here are unstandardized betas, adjusted for clustering within study, as well as for baseline age, gender, and years of education. The betas represent *Z*-score intercepts and yearly change (stage and time × stage interactions). ^a^Compared to amyloid-negative group

*Abbreviations*: *IADL* instrumental activities of daily living, *95%CI* 95% confidence interval

**Fig. 1:**
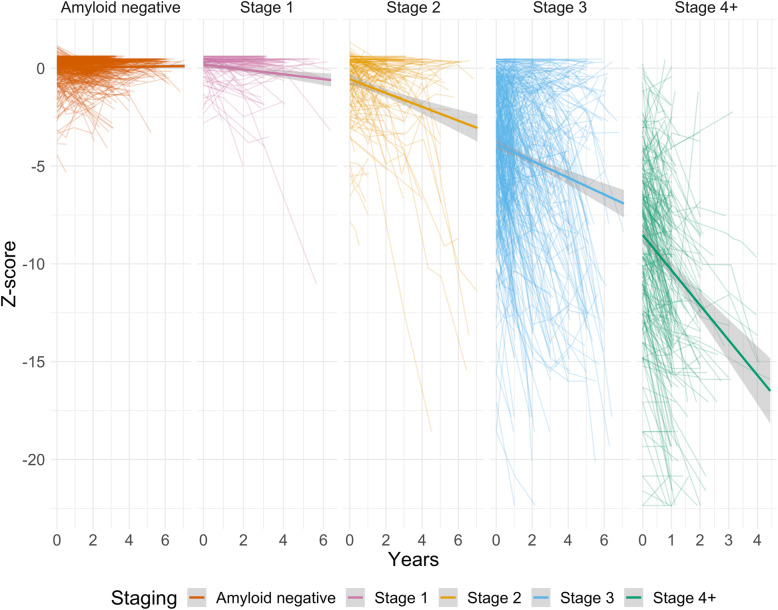
Individual and group average trajectories per clinical stage for the global IADL *Z*-scores. The trajectories show that, at the group level, there is no decline in amyloid-negative individuals, but it does appear to be present in the earliest AD stages, and it increases with each subsequent stage. A one-unit change in the *Z*-score represents one standard deviation in the amyloid-negative group

We found that, as a group, individuals in stage 1 showed a small, non-significant decline in IADL functioning over time (*B* = − 0.12, 95%CI = [− 0.37, 0.13], *p* = .342). The rate of decline was only marginally larger than in the amyloid-negative group, and this difference was also not significant. Individuals in stage 2 declined significantly (*B* = − 0.32, 95%CI = [− 0.55, − 0.09], *p* = .007), as did individuals in stages 3 (*B* = − 1.06, 95%CI = [− 1.27, − 0.85], *p* < .001) and 4+ (*B* = − 1.93, 95%CI = [− 2.19, − 1.67]). Moreover, when comparing the slopes in all stages and amyloid-negative controls with each other, there was a significant time × stage interaction for all stages, except the first stage (Table 3). The rate of decline accelerated with each successive stage (stage 1, − 0.12; stage 2, − 0.32; stage 3, − 1.06; stage 4, − 1.93; Table 3), compared to amyloid-negative individuals. Figure 1 displays the individual trajectories and group slopes of IADL decline for each stage. As can be seen in Fig. 1, there was a large variability in slopes between individuals in the AD continuum.

We additionally investigated the trajectories for community-based and memory clinic study cohorts and found that the results were largely similar, except that in community-based studies, the decline observed in stage 2 was not significantly different from the change in amyloid negatives. The results from these analyses can be found in the Supplementary Material.

### Activity-specific trajectories

**Fig. 2:**
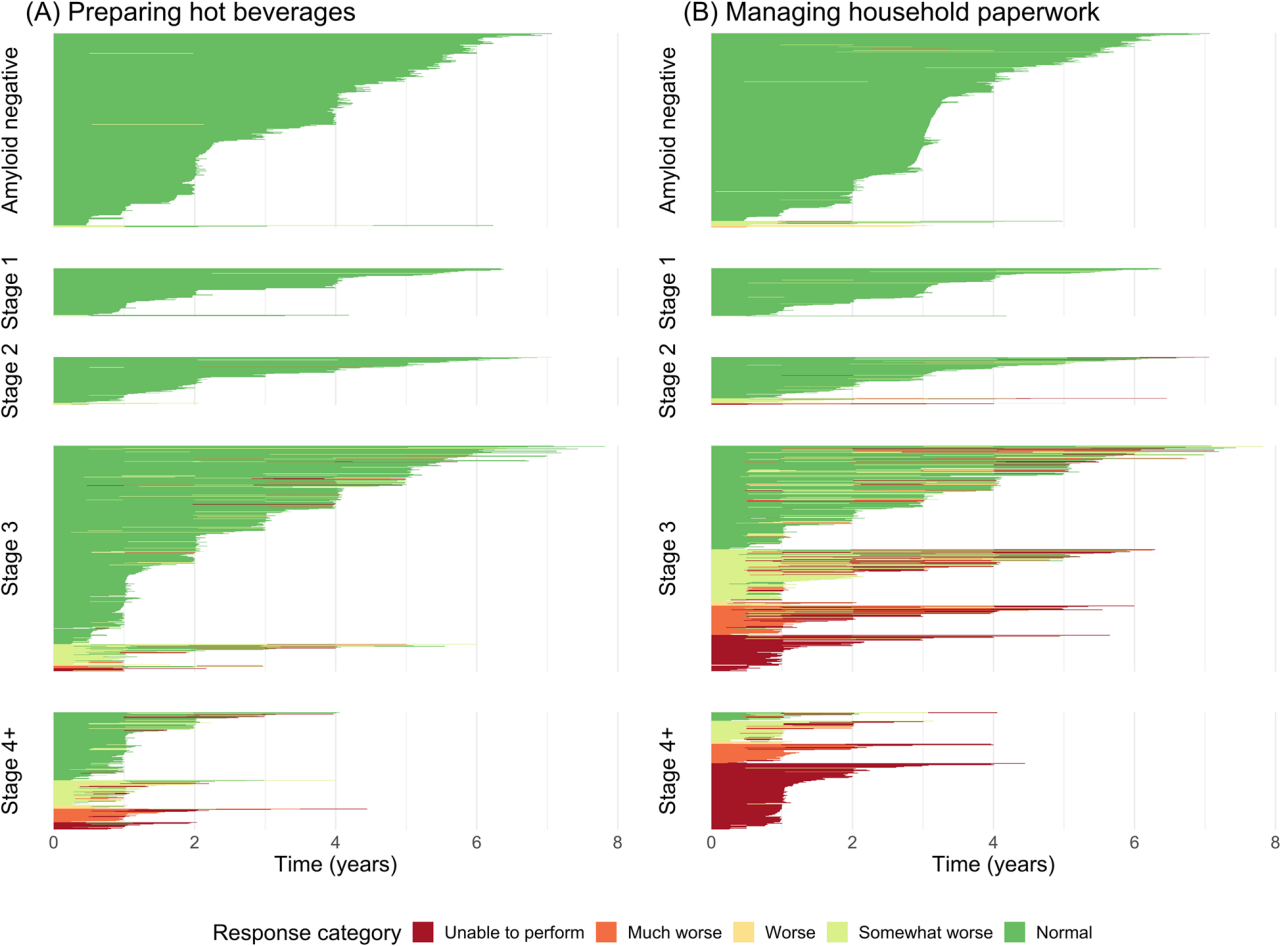
Individual response categories on two pooled activities: **a** “preparing hot beverages” and **b** “managing the paperwork”. Each horizontal line represents an individual (on the *y*-axis), with longer lines representing longer follow-up (time in years on the *x*-axis). The lines are colored based on the level of difficulty the individual had over the course of their follow-up, ranging from dark green (normal performance) to dark red (unable to perform). Individuals are grouped by NIA-AA clinical stage

Both the relatively easy “preparing hot beverages” (estimate − 0.68, 95%CI = [− 0.80, − 0.57]) and the more complex “managing paperwork” (estimate − 0.66, 95%CI = [− 0.75, − 0.57]) showed a similar, significant decline in the amyloid-positive group as a whole. Compared to the amyloid-negative group, individuals in stage 2 declined significantly faster on preparing hot beverages (*p* < .001), whereas on managing paperwork, even individuals in stage 1 declined significantly faster (*p* = .007). For both activities, there were no significant differences in rate of decline between stages 2 and 3. Those in stage 4+ declined the fastest on both activities. Individual item responses are shown in Fig. 2.

## Discussion

We demonstrated that the decline in daily functioning accelerates as AD progresses along the continuum from preclinical to symptomatic. This decline was distinct from the functional change observed in amyloid-negative, cognitively normal individuals. Furthermore, our data suggest that more complex activities, such as managing the paperwork, are especially sensitive to the earliest cognitive changes, showing a decline in the early prodromal stage.

Functional impairment has long been considered a defining feature of the transition from mild cognitive impairment to the dementia stage of AD [44, 45]. Accumulating evidence over the past decade shows that difficulties in cognitively complex activities may be seen in cognitively normal individuals who later progress to dementia [46–48]. This might indicate that these individuals have a lower level of functioning to start with, or it might suggest a decline in the pre-dementia stages. These findings have not previously been investigated in the context of the newly proposed NIA-AA stages. The staging criteria propose that detectible but mild functional impairment may be found in stage 3 and beyond, when performance on objective cognitive tests becomes impaired [1]. We present evidence that decline in functional impairment may already be present in earlier stages. The decline we observed in stage 1 did not differ from the change in amyloid-negative individuals and may have been too subtle to be distinguished from normal aging-related decline in everyday functioning. Others have previously stated that sensitive measures of cognitive and functional impairment are needed to monitor disease progression in early stages, or to evaluate drug effectiveness in the context of AD clinical trials [49, 50]. More convincingly, we observed a decline in stage 2, before cognitive decline can be objectively measured using traditional cognitive tests. When compared to cognitively normal amyloid-negative individuals, there was a faster decline in all subsequent stages from stage 2 onward. In community-based studies, individuals in stage 2, which corresponds approximately to the concept of subjective cognitive decline (SCD), did not decline at a significantly different rate than amyloid-negative, cognitively normal individuals. It is possible that stage 2 individuals who have not visited a memory-clinic may be in some way different from those who have. Slot and colleagues [3] have previously shown that people with SCD who visited a memory clinic had an increased risk of progressing to dementia, compared to those who were included in community-based studies.

Our findings demonstrate that functional decline co-occurs with the earliest changes in cognition in the context of AD, revealing the importance of assessing daily functioning in addition to cognitive functioning, particularly in early stages. Decline in cognition is assumed to cause functional impairment, not vice versa. However, many frequently used cognitive measures might not be sensitive enough to detect subtle cognitive changes [51–53]. Our results justify combining sensitive IADL measures with sensitive cognitive tests for detecting such changes. Study partner-reported functional questionnaires have additional advantages in that they are easy to administer, have good ecological validity, and are strongly related to quality of life [18, 54]. As such, our findings implicate an important benefit of including the measurement of everyday functioning in early AD stages for the evaluation of disease progression and potential intervention effectiveness, in addition to providing potential starting points for early non-pharmacological interventions targeting cognitive functioning.

### Limitations

This study had a few limitations. Three questionnaires assessing slightly different aspects of cognitively complex everyday functioning and in reference to differing time frames were combined, and total scores were placed on a single scale by computing *Z*-scores for each instrument and merging them into a single score. Of all included activities, only a handful overlapped between all three questionnaires. Overall, however, they provide information about the same construct: higher-order cognitive functioning in everyday life, which was partly evidenced by the high correlation between two of the three measures. This justifies the combination of total scores into a single functional measure. Future undertakings could adapt a more sophisticated linking method, e.g., by using item response theory, giving more weight to questionnaires with favorable psychometric properties. A second limitation was that amyloid positivity was assessed using different techniques (i.e., PET and CSF) and using local cut-offs, so that an individual found positive in one cohort might not have been found positive in another. The average follow-up time was approximately 3 years. For the early stages (1 and 2), 3 years is a relatively short period of time, as preclinical AD duration is estimated to be about 10 years [55]. Further, we did not include longitudinal assessment of cognition and can therefore not be sure whether participants progressed from one clinical stage to the next. In consequence, it should be taken into account that our trajectories of change might not reflect each stage’s entire duration. Finally, our study sample was comprised of convenience samples with relatively highly educated and mostly Caucasian participants. This has potentially caused a sample bias, and our findings may therefore not be directly applied to the global population.

### Strengths

An important strength of this study was the large number of amyloid-positive individuals with a large age range and representing the entire AD continuum, who were followed over time and recruited in different study settings from both the USA and the Netherlands. By combining data from different cohorts, we aimed to overcome at least in part the sample bias. We ran sensitivity analyses (in Supplementary Material) and found that results were robust when removing either one of the cohorts, suggesting that the results are not driven by a single measure or cohort, supporting the robustness of our findings. Another strength was our approach to define clinical stages of severity, by using a careful operationalization of the NIA-AA clinical staging scheme and grouping individuals into four different clinical stages, which is a more refined method than relying solely on diagnostic status [56]. Additionally, IADL functioning as determined by the three questionnaires was not part of the staging criteria used in the current study, which has been a confound in many previous studies which divided groups into MCI and dementia. However, it must be noted that the staging was not completely independent of IADL as a construct, and that clinicians who determined disease symptom severity may not always have been blinded to the IADL scores, which may have influenced their classification. Our inclusion of an amyloid-negative comparison group indicates that the decline in IADL is disease specific and not a general aging effect.

Future research should include the other two major components of the NIA-AA model of AD, tau and neurodegeneration, to further investigate the relationship between function and AD pathology. Future studies should also incorporate and combine longitudinal clinical staging, so the continuous progression of cognitive and functional performance along the AD clinical spectrum can be investigated. Because functional impairment is not unique to AD, future research should replicate our study in other neurodegenerative diseases, to investigate the relationship between other types of neurodegeneration and IADL functioning. Furthermore, as we noticed a lot of intra- and inter-individual variability in the change over time, it would be interesting to delve into these individual differences in future studies. Finally, we currently do not know when changes in functioning actually affect a person’s ability to function independently. As such, investigating the clinical meaningfulness of these changes would be an important future endeavor.

## Conclusion

To conclude, our findings suggest that increased difficulties with cognitively complex everyday activities may constitute a useful marker of early cognitive decline, in the pre-dementia stage of AD. Thus, the assessment of these complex activities may provide valuable information about the severity of cognitive symptoms, especially when measured longitudinally. Incorporating IADL measures alongside cognitive tests would allow for within-individual everyday decline to be gauged in a cost- and time-effective way. We therefore recommend including a measure of functional difficulties in clinical trials at the stage of preclinical AD, as well as in clinical practice.

## Supplementary information

**Additional file 1.**

## Data Availability

The data and materials used in this study can be made available by the corresponding author upon request.
