# Supplementary material for: Decline in cognitively complex everyday activities accelerates along the Alzheimer’s disease continuum
Source: Alzheimers Res Ther. 2020 Oct 29;12:138. doi: 10.1186/s13195-020-00706-2 (PMC7597034; doi:10.1186/s13195-020-00706-2)
Supplement: Supplementary file 1 — Additional file 1. [file 13195_2020_706_MOESM1_ESM.docx]

# Supplementary Material

## Cohort descriptives

In addition to the sample characteristic table included in the main text, we provide descriptives of each of the included cohorts in the table below.

Table 1 Cohort sample descriptives

|  | **HABS** | **ADNI** | **NACC** | **ADC** | **EMIF-AD** | **EMIF-90+** |
| --- | --- | --- | --- | --- | --- | --- |
| N | 259 | 829 | 201 | 178 | 73 | 15 |
| Age | 73.64 ± 6.1 | 74.20 ± 7.1 | 69.69 ± 7.9 | 66.12 ± 6.8 | 67.57 ± 6.0 | 92.08 ± 1.6 |
| Female, n (%) | 156 (60) | 390 (47) | 90 (45) | 73 (41) | 54 (74) | — |
| Education years | 15.93 ± 3.0 | 16.21 ± 2.7 | 16.35 ± 2.8 | 11.22 ± 2.9 | 11.70 ± 2.6 | 13.60 ± 4.9 |
| MMSE | 29.03 ± 1.1 | 27.57 ± 2.8 | 15.57 ± 3.6 | 22.09 ± 4.8 | 29.05 ± 1.2 | 28.73 ± 1.4 |
| White race, n (%) | 215 (82) | 760 (92) | 186 (93) | — | — | — |

Data are presented as mean ± standard deviation, unless otherwise indicated. “—” denotes that data was unavailable. MMSE scores were taken from the baseline assessment.
Abbreviations: ADC, Amsterdam Dementia Cohort; ADNI, Alzheimer’s Disease Neuroimaging Initiative; EMIF, European Medical Information Framework; HABS, Harvard Aging Brain Study; MMSE, Mini-Mental State Examination; NACC, National Alzheimer’s Coordinating Center.

## Sensitivity analyses

We ran sensitivity analyses to investigate whether our findings were independent of study cohort and type of recruitment setting. Models in which we excluded one of the study cohorts are shown in Table 2. In general, the interpretation of the effects remained the same across all models. The estimates without ADNI are somewhat different, likely because ADNI was the largest sample in our study and comprised more than half the sample size. Without ADNI the change in IADL functioning in stage 2 was not different from the change in IADL functioning in amyloid negative individuals.

Table 2 Sensitivity analyses excluding each cohort once

| **Slopes** | **Without HABS** | | **Without ADNI** | | **Without NACC** | | **Without ADC/EMIF** | |
| --- | --- | --- | --- | --- | --- | --- | --- | --- |
|  | **B** | **95%CI** | **B** | **95%CI** | **B** | **95%CI** | **B** | **95%CI** |
| Amyloid negative | -0.046 | [-0.158, 0.065] | -0.105 | [-0.203, -0.008] | -0.050 | [-0.115, 0.014] | -0.051 | [-0.123, 0.021] |
| Amyloid positive | -1.089 | [-1.178, -1.002] | -0.872 | [-1.002, -0.745] | -0.908 | [-0.978, -0.840] | -0.996 | [-1.076, -0.919] |
| Stage 1 | -0.155 | [-0.396, 0.086] | -0.135 | [-0.352, 0.081] | -0.123 | [-0.271, 0.026] | -0.141 | [-0.299, 0.018] |
| Stage 2 | -0.351 | [-0.542, -0.160] | -0.213 | [-0.429, 0.002] | -0.342 | [-0.473, -0.212] | -0.306 | [-0.449, -0.163] |
| Stage 3 | -1.056 | [-1.163, -0.949] | -0.828 | [-1.042, -0.614] | -1.009 | [-1.092, -0.926] | -1.076 | [-1.169, -0.983] |
| Stage 4+ | -1.949 | [-2.145, -1.753] | -2.013 | [-2.286, -1.740] | -1.654 | [-1.838, -1.469] | -2.036 | [-2.242, -1.830] |

Abbreviations: 95%CI = 95% confidence interval

Shown here are unstandardized betas, adjusted for baseline age, gender, and years of education. The betas represent yearly change in the combined Z-score of the three IADL instruments.

^†^ different from amyloid negative controls

Stratified models for community-based and memory clinic cohorts are in Table 3. In the community-based cohorts, the rate of decline in IADL functioning is very similar between stages 1 and 2, whereas the rate of decline is faster in stage 2 than in stage 1 in the memory-clinic cohorts. The effects in the other stages are similar between community-based and memory clinic studies.

Table 3 Sensitivity analyses stratified for community-based vs. memory clinic study cohorts

|  | **Community-based** | | **Memory clinic** | |
| --- | --- | --- | --- | --- |
| **Slopes** | **B** | **95%CI** | **B** | **95%CI** |
| Amyloid negative | -0.07 | [-0.19, 0.04] | -0.01 | [-0.11, 0.09] |
| Amyloid positive |  |  |  |  |
| Stage 1 | -0.14 | [-0.38, 0.10] | -0.14 | [-0.37, 0.09] |
| Stage 2 | -0.13 | [-0.41, 0.15] | -0.40^†^ | [-0.58, -0.21] |
| Stage 3 | -1.21^†^ | [-1.60, -0.81] | -1.02^†^ | [-1.12, -0.93] |
| Stage 4+ | -2.55^†^ | [-3.01, -2.08] | -1.67^†^ | [-1.87, -1.47] |

Abbreviations: 95%CI = 95% confidence interval

Shown here are unstandardized betas, adjusted for baseline age, gender, and years of education. The betas represent yearly change in the combined Z-score of the three IADL instruments.

^†^ different from amyloid negative controls

Finally, we ran sensitivity models using each of the three instruments separately, which can be found in Table 4. The FAQ and ECog show similar effects in all stages, with increasing rates of decline in each subsequent stage, and a significant difference in rate of decline between stage 2 and amyloid negatives. The A-IADL-Q shows similar effects as well, however, these are not significant, possibly due to the smaller sample sizes. The estimate of the slope in stage 1 is based on just 4 individuals and should hence be interpreted with caution.

Table 4 Sensitivity analyses for each separate IADL instrument,

|  | **FAQ** |  | **ECog** |  | **A-IADL-Q** |  |
| --- | --- | --- | --- | --- | --- | --- |
| **Slopes** | **B** | **95%CI** | **B** | **95%CI** | **B** | **95%CI** |
| Amyloid negative | -0.01 | [-0.16, 0.14] | -0.04 | [-0.08, 0.01] | -0.30 | [-0.57, -0.04] |
| Amyloid positive |  |  |  |  |  |  |
| Stage 1 | -0.16 | [-0.47, 0.15] | -0.11 | [-0.21, -0.02] | 1.04 | [-0.48, 2.57] |
| Stage 2 | -0.43^†^ | [-0.70, -0.16] | -0.25^†^ | [-0.34, -0.16] | -0.41 | [-0.80, -0.02] |
| Stage 3 | -1.46^†^ | [-1.60, -1.31] | -0.53^†^ | [-0.59, -0.48] | -0.67 | [-0.94, -0.40] |
| Stage 4+ | -2.62^†^ | [-2.92, -2.31] | -0.83^†^ | [-1.00, -0.67] | -1.56^†^ | [-1.89, -1.22] |

Abbreviations: A-IADL-Q, Amsterdam Instrumental Activities of Daily Living Questionnaire; ECog, Everyday Cognition; FAQ, Functional Activities Questionnaire; 95%CI = 95% confidence interval

Shown here are unstandardized betas, adjusted for baseline age, gender, and years of education. The betas represent yearly change in the Z-score of each IADL instrument.

^†^ different from amyloid negative controls

## Amyloid classification procedures

PET data were available for ADNI, HABS and both EMIF-AD studies, while both CSF and PET were used in NACC and ADC. Amyloid binding was measured in HABS using Pittsburgh compound-B (PiB), florbetapir in ADNI, and one of PiB, flutemetamol, florbetapir, or florbetaben in the ADC and EMIF-AD studies. Amyloid positivity was determined in HABS on distribution volume ratio of mean uptake in frontal, lateral parietal and temporal, and retrosplenial regions (cutoff ≥ 1.20). In ADNI, amyloid positivity was based on standard uptake value ratios of mean uptake in four cortical regions, normalized to the whole cerebellum uptake (cut-off value ≥ 1.10). In both EMIF-AD studies, amyloid positivity was determined by consensus on visual read of PET scans by multiple independent physicians. In the ADC, PET scans were visually rated by an experienced nuclear medicine physician who was blinded to clinical information. In the NACC cohort, amyloid positivity in PET or CSF was determined using each center’s local cutoffs. CSF amyloid positivity was determined as being below the ADC’s cutoff of 813 pg./mL. Where CSF and PET were available for the same individual, PET results were favored in case of disagreement.
